# Solvent-Free Mechanochemical Synthesis of High Transition Biphenyltetracarboxydiimide Liquid Crystals

**DOI:** 10.3390/molecules26103035

**Published:** 2021-05-19

**Authors:** Jehan Y. Al-Humaidi, Siham A. Alissa, Kanubhai D. Katariya, Khulood A. Abu Al-Ola, Mohamed Hagar, Khaled D. Khalil

**Affiliations:** 1 Department of Chemistry, College of Science, Princess Nourah bint Abdulrahman University, Riyadh 11671, Saudi Arabia; Jyalhamidi@pnu.edu.sa (J.Y.A.-H.); saalissa@pnu.edu.sa (S.A.A.); 2 Department of Chemistry, Faculty of Science, The Maharaja Sayajirao University of Baroda, Vadodara 390002, India; kanukatariya-chemphd@msubaroda.ac.in; 3 Chemistry Department, College of Sciences, Al-Madina Al-Munawarah, Taibah University, Al-Madina 30002, Saudi Arabia; Kabualola@taibahu.edu.sa; 4 Chemistry Department, College of Science, Taibah University, Al-Madinah Almunawrah, Yanbu 46423, Saudi Arabia; khd.khalil@yahoo.com; 5 Chemistry Department, Faculty of Science, Alexandria University, Alexandria 21321, Egypt; 6 Chemistry Department, Faculty of Science, Cairo University, Giza 12613, Egypt

**Keywords:** mechanochemical synthesis biphenyltetracarboxydiimide liquid crystals, high transition temperature, smectogenic diimide, DFT calculations, conformational investigation

## Abstract

A series of high temperature alkyl and alkoxy biphenyltetracarboxydiimide liquid crystals have been prepared under ball mill method using solvent-free mechanochemical approach. The thermal properties of the prepared compounds were investigated by deferential scanning calorimetry (DSC) measurements and the textures were identified by polarized optical microscope (POM). The compounds showed smectic mesomorphic behaviour. The results showed the increasing nature of transition temperature Cr-SmC with chain length with increments of the SmC mesophase range. However, the mesophase range of the SmA was decreased with the terminal chain length either for the alkyl or alkoxy terminal groups. Moreover, the DFT theoretical calculations have been conducted give a detailed projection of the structure of the prepared compounds. A conformational investigation of the biphenyl part has been studied. A deep illustration of the experimental mesomorphic behaviour has been discussed in terms of the calculated aspect ratio. A projection of the frontier molecular orbitals as well as molecular electrostatic potential has been studied to show the effect of the polarity of the terminal chains on the level and the gap of the FMOs and the distribution of electrostatic charges on the prepared molecules.

## 1. Introduction

In recent years, the design and development of thermotropic liquid crystalline materials comprising heteroatoms/heterocyclic rings have been potentially pursued for the preparation of advanced functional materials. Stress-free modification of molecular shapes and molecular properties of a newly prepared compounds including polarity, phase structure, geometry and can be varied by the inclusion of heteroatoms/heterocyclic unit and hence heterocyclic-based liquid crystalline compounds (LCs) has gained attraction of researchers [1,2,3]. As a matter of fact, because of these features they have been used for a number of photochemical and optical applications, organic transistors [4], including optical signal processing and storage [5], organic photovoltaic devices [6,7] and switching ferroelectric materials [8]. Over the last few years, a noticeable number of thermotropic liquid crystalline compounds having core units consist of various heterocycles have been designed, prepared and characterized [9,10,11,12,13,14].

The molecular order in the phases of liquid crystal materials is largely governed by the structure of mesogenic core and, as a result, any significant change in mesomorphic properties of a liquid crystalline compound largely depends on varying the core structure of a mesogenic compounds. Introduction of electronegative heteroatoms (such as nitrogen, oxygen, and sulphur)/heterocyclic rings into the rigid core of π-conjugated systems often develops powerful polar induction by producing reduced symmetrical structure of the complete molecule [5]. Highly π-conjugated mesogenic heterocyclic compounds are displaying enhanced applications in organic photonics [1,15]. Functional molecular properties arise particularly from the heteroatoms/heterocycle’s capability to impart lateral and/or longitudinal dipoles reliant on the shape of a molecule [16,17]. In addition, it has been observed that the presence of heteroatoms impact the type of mesophase, the phase transition temperatures, dielectric and other properties of the mesogenic compound because of the intermolecular interactions they do possess [18].

Over the past few years, thiadiazoles [19,20], oxadiazoles [21,22,23], benzoquinoxalines [24,25], benzimidazoles [26,27], benzoxazoles [28,29], and diimides [30,31,32] have recently been explored as acceptor entities to obtain diverse types of architectural units and liquid crystalline properties. Among heterocycles, Isoindoline-1,3-dione (phthalimide), a significant class of compounds, has attracted significant attention and remained a centre of interest among researchers in the fields such as organic chemistry, medicinal applications, analytical and coordination chemistry. It has been reported that phthalimide (-CO-N(R)-CO-) possess a wide range of biological activities including anti-microbial, anti-tumour and DNA cleaving activities because of the hydrophobic nature of the phthalimide which enhances their ability to cross various biological membrane [33,34]. The compounds containing phthalimide heterocycle as a part of their structure also shows high fluorescence yields, photo stability, strong absorption and emission in the ultraviolet-visible region, they are also employed in the applications of molecular sensors for the cations and anions recognition [35]. Presence of π-π staking interactions in compounds possessing phthalimide make them favourable materials then electron withdrawing materials for organic electronics such as OLEDs [36]. Apart from all above mentioned applications, the chemistry of imide derivatives has also been explored for the synthesis of liquid crystalline compounds and studied for their mesomorphic properties with a variety of structural changes [35,37,38,39,40]. 

Continuing our interest [9,10,11] in finding a facile, efficient and green method for preparation of nitrogen heterocycles, herein, the aim of this work is to investigate an efficient green ball milling solvent-free synthesis of a series of benzimidazoles in a good yield. The outcome of a chemical reaction in a ball mill mainly depends on the amount of energy that is supplied. Several reaction parameters directly influence this energy input. Some of these parameters are rotation frequency, milling balls weight and milling time.

In addition, this work aims to prepare a series of high temperature alkyl and alkoxy biphenyltetracarboxydiimide liquid crystals. Complete investigations of thermal optical properties will also be another aim of this paper. Finally, continuing our interest [41,42,43,44,45,46,47] in conducting the experimental results with density functional theory (DFT) theoretical calculations is another goal. In addition, the pursuit of our interest [48,49,50,51,52,53] as well as other researchers [54,55,56,57,58,59,60] in finding a facile, efficient and green method for preparation of nitrogen heterocycles, herein, another aim of this work is to investigate as a comparative study of use of the solvent-free synthesis of 3,3′,4,4′-Biphenyltetracarboxy-*N*,*N′*-bis-(alkyl or alkoxyphenyl)diimide, **2a**–**2f** using the mechanochemical approach using our previous method [52].

## 2. Results and Discussion

### 2.1. Methods of Preparation

The preparation of 3,3′,4,4′-Biphenyltetracarboxy-*N*,*N*′-bis-(alkyl or alkoxyphenyl)diimides, **2a**–**2f** was carried out using two methods. The first was the traditional process, which was carried out in DMF under reflux for six hours. The other approach is to use a ball mill in a solvent-free environment with a very fast reaction time (15 min). The reaction went smoothly, resulting in excellent yields of the final product (95–99%). Under our previous optimum conditions, the molar quantities of alkyl and alkoxyanilines and 3,3′,4,4′-biphenyltetracarboxylic anhydride were milled [52]. The reactants were placed in 250 cm^3^ stainless steel vials containing 56.6 g of stainless steel balls (4 (12.7 mm), 4 (6.3 mm), and 4 (3.2 mm)), and the reaction was carried out at 20 Hz for 15 min to obtain quantitative quantities of the final products.

The weight of the milling balls is one of the technological parameters that describe the variables that can affect the yield of the reaction. The yield of the reaction product increased as the weight of milling balls used increased, according to the results of Table 1. After 20 min of milling, only 60–65% of the product produced with the smallest 14.4 g of milling balls weight, while, the heaviest 56.60 g raises the total yield of the reaction to 95–99%.

Milling conditions were optimized in order to achieve high yields of the product in a short reaction period. To complete the reaction and prevent by-products, the reaction time needed to be optimized. Taking of the reaction mixture at different times allowed us to assess how far the reaction had progressed (Table 2). When the reaction milling for 15 min milling time was used, the maximum yield of 98% was obtained. The longer reaction time (20 min) was not preferred, as the yield was reduced to 95 percent instead of 98 percent for the shorter time (15 min).

### 2.2. Mesomorphic Properties

**Figure 1:**
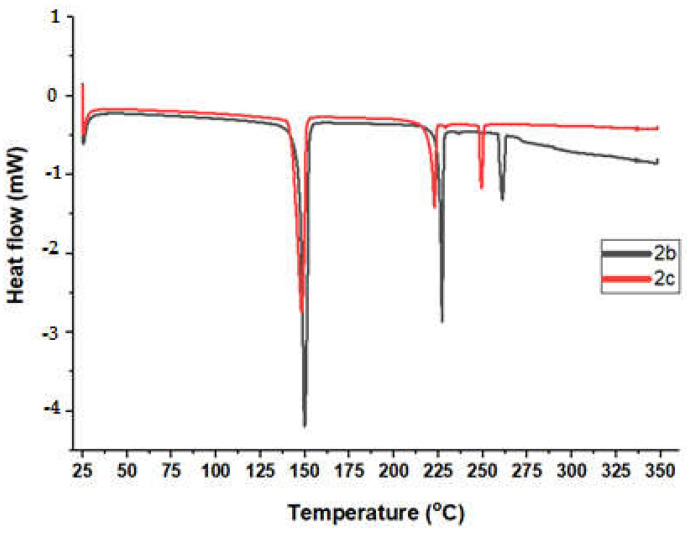
DSC thermogram of new compounds **2b**, and **2c** recorded at a rate of 10 °C/min during heating cycle.

The mesomorphic properties of prepared mesogens **2a**–**2f** were investigated by DSC measurements during heating cycles and subsequently the textures of the new compounds were observed by POM. The obtained DSC data (transition temperatures and enthalpies) are summarized in Table 3 and Figure 1.

**Figure 2:**
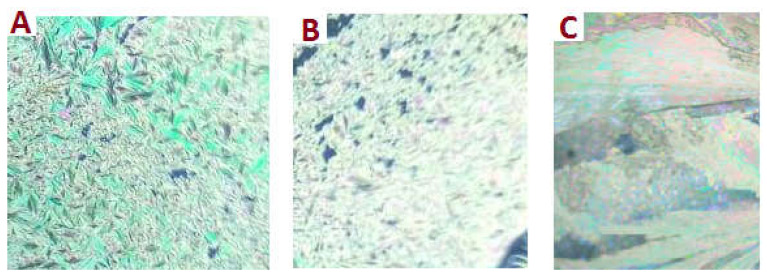
Optical photomicrographs under polarized optical microscopy (POM) of the compound **2c** during the cooling scan. (**A**) Smectic-A mesophase at 267 °C, (**B**) Smectic-C mesophase at 220 °C and (**C**) Crystal phase at 90 °C.

As can be seen from Table 3, all the compounds demonstrated enantiotropic mesomorphic properties of smectic A (SmA) and smectic C (SmC) mesophases and their endotherms have been characterised by the crystal-mesophase-isotropic transitions taking place beyond the melting temperatures observed during the heating and cooling cycles. These transitions have also been supported by the enthalpy values of the corresponding compounds. The phase changes between the solid and liquid crystalline materials were evidently distinct by the presence of sharp peaks in the DSC thermograms; however, the small enthalpy variations characterizing the transitions from LC phase to the isotropic liquid. The DSC thermograms for **2b** and **2c** upon heating scans are presented (Figure 1). Three endotherms have been observed for **2c** on heating and cooling cycles, respectively, which were associated with the crystal-to-smectic C (Cr-to-SmC) and smectic C-to-smectic A (SmC–SmA) and smectic A to isotropic transitions (SmA-to-I) (Figure 1). An intense peak at 149.8 °C in the heating cycle corresponds to the Cr-SmC transition. On the contrary, the SmC–SmA and SmA–isotropic transitions were distinguished by small peaks at 227.1 °C and 262.3 °C correspondingly. Upon cooling the isotropic liquid of **2c**, the presence of the smectic A phase was identified based on the appearance of Smectic A texture at 267 °C (Figure 2A). The smectic A phase were identified by the coexistence of fan-shaped optical textures and homeotropic textures [14]. On continuing the cooling of **2c**, the smectic A phase transits slowly to a SmC phase, characterizing a SmA–SmC transition. Further cooling of these compounds leads to a broken fan-shaped SmC texture at 220 °C and finally crystallized at 145 °C (Figure 2B).

**Figure 3:**
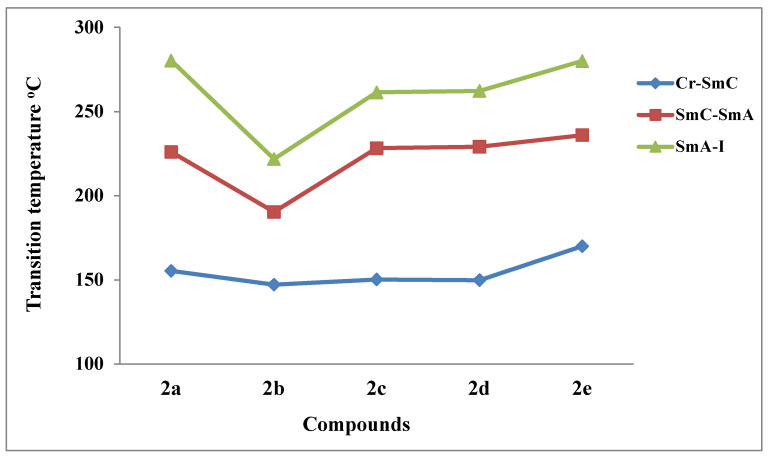
Dependence of mesomorphic transition temperatures on terminal alkoxy chain length.

**Figure 4:**
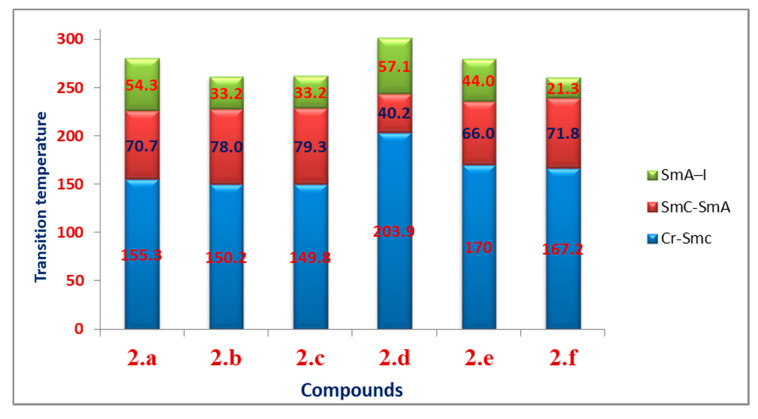
Graphical representation of dependence of mesomorphic phase ranges on the terminal chain length of the new compounds.

The obtained transition temperatures, as a function of alkyl chain length were plotted as presented in Figure 3. The results showed the increasing nature of transition temperature Cr-SmC as we go from **2a** (C10) to **2c** (C14). While for **2f** (OC16) the transition temperature for SmC to SmA transition decreases as compared to **2c**. It was also observed that the transition temperatures for SmC–SmA phase transitions were also increases as we go from **2a** to **2b**. The dependence of mesophase transition temperatures as a function of the terminal alkoxy substituents are presented in Figure 4. It has been observed that as we go from **2a** to **2c** the phase range of Sm A increases while that of range of Sm C phase increases from **2a** to **2b** and again decreases for **2c**. For compound **2f** with -OC_16_H_33_ substitution, the phase ranges Sm A and Sm C decreases that **2b** (-C_12_H_24_) and **2c** (-C_14_H_28_).

### 2.3. DFT Molecular Structure 

**Scheme 1:**
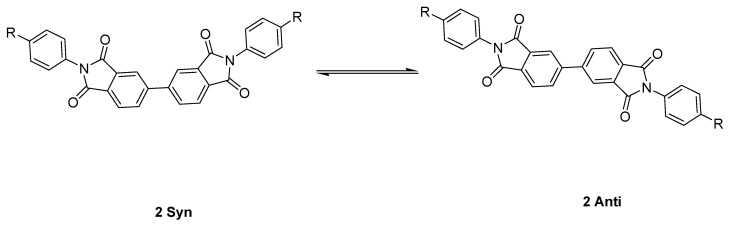
Proposed conformers of 3,3′,4,4′-Biphenyltetracarboxy-*N*,*N*′-bis-(alkyl or alkoxyphenyl)diimides.

The prepared diimides were assumed to exist in two conformers, (syn), (anti) according to the orientation of the biphenyl groups, Scheme 1.

**Figure 5:**
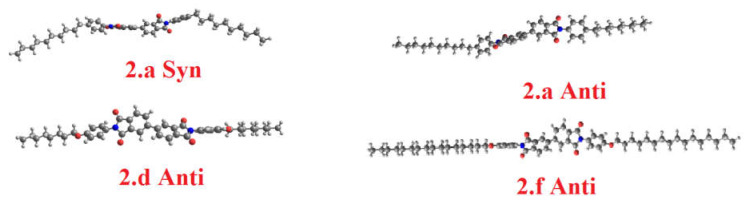
Calculated molecular geometries of Syn conformer of **2a** and the anti-conformers of **2a**, **2d** and **2f**.

The optimized molecular geometries of the prepared compounds have been investigated using DFT calculations at basis set B3LYP 6-311G (d,p). The calculated optimized geometrical structures were calculated in the gas using Gaussian 9. All compounds were minimized by the estimation of their molecular structural optimization to find their minimum-energy structure. Additionally, the optimization process was executed to discover the geometric structure for the minimum energy of the conformations, where the atoms, the bond lengths, and the bond angles of the compounds were moved until new minimum energy of a geometrical structure is established which designated as convergence. The absence of imaginary frequencies is an evidence of the geometrical stability of all H-bonded complexes. Figure 5 shows the optimized geometrical structures of both conformers of compound **2a**, and the anti-conformers of compounds **2d** and **2f**.

Figure 5 emphasizes that the syn isomer is less planar than the anti-one of the same alkyl chain length. Moreover, the alkoxy derivative is more planar than that of the alkyl derivative and this could be attributed to the planar conjugation of the mesomeric RO group with respect to the hyperconjugated R one. It is worth mentioning here that since the calculated molecular geometries in the gaseous state, the presence of the liquid crystalline compounds in the condensed mesophases, the lowest energy may be different and the more elongated species are preferred [9].

The thermodynamic parameters were estimated by DFT calculations applying the same method at the same set for both conformers of the diimides of **2a.** The thermodynamic results tabulated in Table 4 showed that the anti-conformer is more stable than that of its conformer syn isomer by 0.00136 hartree/particle. The extra stability of the anti-isomer could be explained in terms of the degree of conjugation point of view; however, the small energy difference could be an explanation of their intermolecular interconversion. Furthermore, the presence of the nitrogen atoms in one side of the di-nicotinate base could permit high degree of packing rather than the other. Moreover, the presence of the more planar anti-conformer as a more stable isomer is a good illustration of the more ordered smectic mesophase.

**Figure 6:**
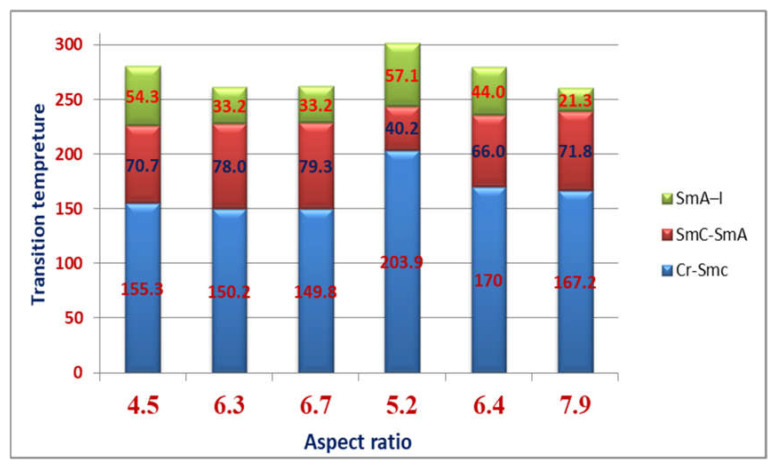
Dependence of the aspect ratio of the prepared compounds with the transition temperature.

**Figure 7:**
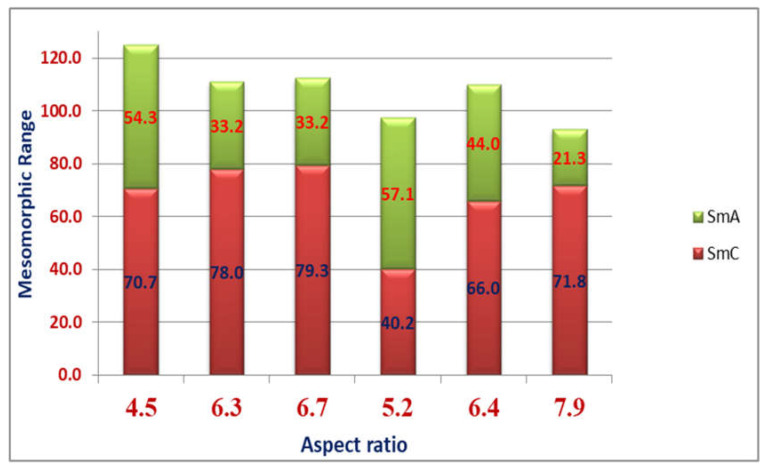
Dependence of the aspect ratio of the prepared compounds with the mesomorphic range.

The relationship between the dimensions of the attached alkoxy and alkyl chain was investigated by studying the effect of the aspect ratio of the molecules with the mesomorphic parameters. Table 5 and Figure 6 and Figure 7 show the dependence of the aspect ratio of the prepared compounds with the transition temperature as well as the mesomorphic range. It is clear that, the mesomorphic behaviour of the polar alkoxy group is somewhat different from that of the nonpolar alkyl group with the same trend. As the chain length increases the aspect ratios increases, however, as we mentioned before, the alkoxy derivatives are more in linear geometrical structure than the alkyl one. The more ordered smectic C mesophase range, the greater the length of the alkyl group, the similar trend of the polar alkoxy group was shown with more degree of enhancement of the SmC mesophase range. On the other hand, the mesomorphic range of the SmA phase decreases with the longer chain lengths either the polar group or the nonpolar one. Such increment in the SmC and decrement in the SmA mesophases could be explained in terms of the degree of the molecular packing of the molecules with the longer chain lengths due to their molecular aggregation. 

Moreover, it is clear from Figure 7 that the SmC mesophase range of the alkyl derivative **2a** (C = 12) is longer than that of the alkoxy derivative of the same length (**2e**). However, the trend is reversed in case of the SmA mesophase range. This could be attributed to the value of the dipole moment of the compounds. The polar alkoxy group enhances a dipole moment higher than that of the alkyl one. The higher dipole moment increases terminal with respect to the parallel interaction, such competitive interactions enhances one phase rather than the other.

### 2.4. Frontier Molecular Orbitals and Polarizability

**Figure 8:**
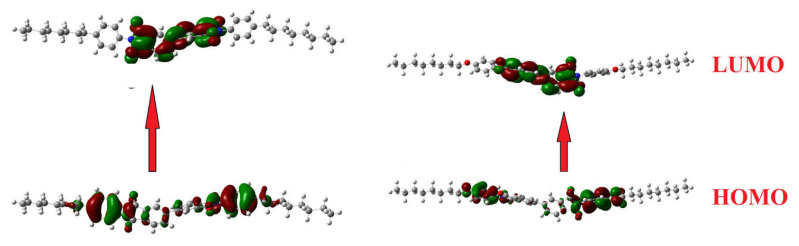
The estimated plots for frontier molecular orbitals of the prepared compounds **2a** and **2d**.

Table 6 and Figure 8 show the estimated plots of frontier molecular orbitals HOMO (highest occupied) and LUMO (lowest unoccupied) of the prepared compounds, **2a** and **2d**. It is obvious from figure, that the electron densities of the sites that shared in the formation of the HOMOs are localized on the aromatic rings with more localization on the phenyl group of the aniline part; however, the LUMOs show no sharing of the aniline rings in the formation of the orbitals. Moreover, it is clear that the polarity of the terminal group has no effect of the location of the electron densities of the FMOs. However, the presence of the polar groups highly impacted the frontier energy gap between the FMOs. The attachment of the polar alkoxy group affects the levels of the frontier molecular orbitals with respect to the nonpolar one of **2a.** This could be explained in terms of the extra conjugation of the aromatic rings in case of the alkoxy group that decreases of the FMOs energy gap. 

## 3. Materials and Methods

**Scheme 2:**
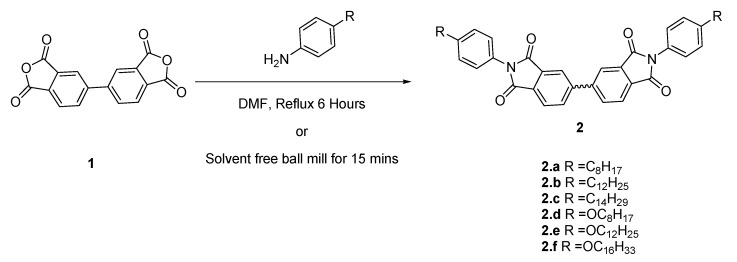
Synthesis of 3,3′,4,4′-Biphenyltetracarboxy-*N*,*N*’-bis-(alkyl or alkoxyphenyl)diimide, **2a**–**2f**.

All chemicals were purchased from Sigma-Aldrich Company (St. Louis, MI, USA). Their purity is higher than 99%. The diimides were prepared according to the following Scheme 2. 

### 3.1. Conventional Method

A mixture of 3,3′,4,4′-biphenyltetracarboxylic anhydride (0.34 mmol, 100 mg) and 4-n-alkylaniline or 4-n-alkoxyaniline (0.85 mmol) was refluxed in DMF for 6 h. The hot solution was left to cool till complete precipitation and then filtered and the crude product was recrystalized from DMF.

### 3.2. Ball Mill (BM) Method

*3,3*′*,4,4*′*-Biphenyltetracarboxy-N-N′-bis-(alkyl or alkoxyphenyl)diimides*, **2a**–**2f** have been synthesized under the best optimal conditions possible using our previous method [1]. A mixture of 3,3′,4,4′-biphenyltetracarboxylic anhydride (0.34 mmol, 100 mg) and 4-n-alkylaniline or 4-n-alkoxyaniline (0.85 mmol) was milled for 15 min in stainless steel vials with 56.6 g of stainless steel balls [4 (12.7 mm), 4 (6.3 mm), 4 (3.2 mm)] at a 20 Hz frequency. To obtain pure compounds, the obtained paste was purified by recrystallization twice from hot DMF.

*3,3*′*,4,4*′*-Biphenyltetracarboxy-N-N*′*-bis-(4-n-octylphenyl)diimide*, **2a** [37]. BM Yield = 99%, ^1^H-NMR (CDCl_3_): δ(ppm) = 8.24 (s, 2H, ArH.). 8.1–7.98 (m, 4H, ArH.), 7.54–7.35 (m, 8 H, ArH), 2.7 (t, 4H, CH_2_), 1.65–1.55 (m, 4H, CH_2_), 1.30–1.21 (m, 20H, CH_2_), 0.88 (t, 6H, CH_3_). IR (KBr): 2928 cm^−1^ C-H stretch, 2853 cm^−1^ C-H stretch, l772 cm^−1^ C=O stretch, 1709 cm^−1^ C=O stretch, 1516 cm^−1^ C=C arom. 

*3,3*′*,4,4*′*-Biphenyltetracarboxy-N-N*′*-bis-(4-n-dodecylphenyl)diimide*, **2b**. BM Yield = 98%, ^1^H-NMR (CDCl_3_): δ(ppm) = 8.24 (s, 2H, ArH.). 8.1–7.98 (m, 4H, ArH.), 7.54–7.35 (m, 8 H, ArH), 2.7 (t, 4H, CH_2_), 1.65–1.55 (m, 4H, CH_2_), 1.30–1.21 (m, 36H, CH_2_), 0.88 (t, 6H, CH_3_). IR (KBr): 2928 cm^−1^ C-H stretch, 2853 cm^−1^ C-H stretch, l772 cm^−1^ C=O stretch, 1709 cm^−1^ C=O stretch, 1516 cm^−1^ C=C arom. 

*3,3*′*,4,4*′*-Biphenyltetracarboxy-N-N*′*-bis-(4-n-tetradecylphenyl)diimide*, **2c**. BM Yield = 95%, ^1^H-NMR (CDCl_3_): δ(ppm) = 8.24 (s, 2H, ArH.). 8.1–7.98 (m, 4H, ArH.), 7.54–7.35 (m, 8 H, ArH), 2.7 (t, 4H, CH_2_), 1.65–1.55 (m, 4H, CH_2_), 1.30–1.21 (m, 42H, CH_2_), 0.88 (t, 6H, CH_3_). IR (KBr): 2928 cm^−1^ C-H stretch, 2853 cm^−1^ C-H stretch, l772 cm^−1^ C=O stretch, 1709 cm^−1^ C=O stretch, 1516 cm^−1^ C=C arom. 

*3,3*′*,4,4*′*-Biphenyltetracarboxy-N-N*′*-bis-(4-n-octyloxyphenyl)diimide,* **2d** [37]. BM Yield = 97%, ^1^H-NMR (CDCl_3_): δ(ppm) = 8.24 (s, 2H, ArH.). 8.1–7.98 (m, 4H, ArH.), 7.54–7.35 (m, 8 H, ArH), 4.31 (t, 4H, CH_2_), 1.65–1.53 (m, 4H, CH_2_), 1.33–1.25 (m, 20H, CH_2_), 0.89 (t, 6H, CH_3_). IR (KBr): 2928 cm^−1^ C-H stretch, 2853 cm^−1^ C-H stretch, l772 cm^−1^ C=O stretch, 1709 cm^−1^ C=O stretch, 1516 cm^−1^ C=C arom. 

*3,3*′*,4,4*′*-Biphenyltetracarboxy-N-N*′*-bis-(4-n-dodecyloxyphenyl)diimide,* **2e** [49]. BM Yield = 98%, ^1^H-NMR (CDCl_3_): δ(ppm) = 8.24 (s, 2H, ArH.). 8.1–7.98 (m, 4H, ArH.), 7.54–7.35 (m, 8 H, ArH), 4.34 (t, 4H, CH_2_), 1.65–1.56 (m, 4H, CH_2_), 1.36–1.20 (m, 36H, CH_2_), 0.89 (t, 6H, CH_3_). IR (KBr): 2931 cm^−1^ C-H stretch, 2855 cm^−1^ C-H stretch, l770 cm^−1^ C=O stretch, 1710 cm^−1^ C=O stretch, 1516 cm^−1^ C=C arom. 

*3,3*′*,4,4*′*-Biphenyltetracarboxy-N-N’-bis-(4-n-hexadecyloxyphenyl)diimide*, **2f**. BM Yield = 96%, ^1^H-NMR (CDCl_3_): δ(ppm) = 8.24 (s, 2H, ArH.). 8.1–7.98 (m, 4H, ArH.), 7.54–7.35 (m, 8 H, ArH), 4.23 (t, 4H, CH_2_), 1.65–1.55 (m, 4H, CH_2_), 1.30–1.21 (m, 52H, CH_2_), 0.88 (t, 6H, CH_3_). IR (KBr): 2932 cm^−1^ C-H stretch, 2858 cm^−1^ C-H stretch, l769 cm^−1^ C=O stretch, 1711 cm^−1^ C=O stretch, 1515 cm^−1^ C=C arom. 

## 4. Conclusions

A solvent-free mechanochemical approach was considered as an efficient route for synthesis of a series of high temperature alkyl and alkoxy biphenyltetracarboxydiimide liquid crystals using the ball mill method. The mesomorphic behaviour of a series of alkyl and alkoxy biphenyltetracarboxydiimide revealed smectogenic high temperature liquid crystals. The results showed that direct proportion of the smectic C range and indirect smectic C range with alkoxy chain length. This result was attributed to the higher degree of molecular aggregation at longer chain length which increases the stability of the more ordered smectic C. The conformational analysis of the biphenyl moiety was studied in terms of the DFT calculations to reveal the Syn form stability by 0.00136 Hartree/Particle and there is no effect of the polar chains on the stability. Relationship between calculated aspect ratio of the molecules with the mesomorphic parameters was also studied. The results showed that more ordered smectic C mesophase range increases as aspect ratio increases with more degree of enhancement of the SmC mesophase range for polar alkoxy groups. However, the trend is reversed in case of the SmA mesophase range and this attributed to the higher value of the dipole moment of the compounds. Finally, the FOMs were investigated to reveal that the presence of the polar groups highly impacted the frontier energy gap between the FMOs. This was attributed to the extra conjugation of the aromatic rings in case of the alkoxy group and consequently to the decrease in the FMOs energy gap. 

## Figures and Tables

**Table 1:** Milling of 100 mg of 3,3′,4,4′-biphenyltetracarboxylic anhydride and 174 mg of octyl aniline at 20 Hz for 15 min.

Entry	Balls (g)	No of Balls (mm Diameter)	% Yield
1	14.12	1 (12.7 mm), 1 (6.3 mm), 1 (3.2 mm)	60–65
2	28.27	2 (12.7 mm), 2 (6.3 mm), 2 (3.2 mm)	86–89
3	56.60	4 (12.7 mm), 4 (6.3 mm), 4 (3.2 mm)	95–99

**Table 2:** Milling of 100 mg of 3,3′,4,4′-biphenyltetracarboxylic anhydride and 174 mg of octyl aniline with 56.6 g of balls at 20 Hz.

Entry	Time Min	% Yield
1	5	70
2	10	91
3	15	98
4	20	95

**Table 3:** Transition temperature (°C), associated (enthalpy of transitions in kJ g^−1^).

Compound	R	*T*_Cr–SmC_ (°C) (∆*H*_Cr–SmC_)	*T*_SmC–SmA_ (°C) (∆*H*_SmC–SmA_)	*T*_SmA–I_ (°C) (∆*H*_SmA–I_)
**2a**	-C_8_H_17_	155.3	226.0	280.3
**2b**	-C_12_H_25_	150.2 39.06	228.2 17.59	261.4 9.07
**2c**	-C_14_H_29_	149.8 33.05	229.1 8.36	262.3 2.73
**2d**	-OC_8_H_17_	203.9	244.1	301.2
**2e**	-OC_12_H_25_	170.0	236.0	280.0
**2f**	-OC_16_H_33_	167.2 15.15	239.0 1.47	260.3 1.65

Cr–SmC indicates transition from solid to the SmA phase. SmC–SmA indicates transition from SmC to the SmA phase. SmA–I signifies transition from SmA to isotropic phase.

**Table 4:** Thermal parameters (Hartree/Particle) of both conformers of **2a**.

Parameter	2a Syn	2a Anti
E_corr_	0.942628	0.942357
ZPVE	−2272.327478	−2272.328077
E_tot_	−2272.274203	−2272.275567
H	−2272.273259	−2272.274623
G	−2272.428135	−2272.426559
ΔE	00000	0.00136

Abbreviations; ZPVE: Sum of electronic and zero-point energies; E_tot_: Sum of electronic and thermal energies; H: Sum of electronic and thermal enthalpies; G: Sum of electronic and thermal free energies.

**Table 5:** Dependence of the aspect ratios of the prepared compounds with mesomorphic behaviours.

Compounds	T_C_, Crystal	ΔT Smectic C	ΔT Smectic A	ΔT Total Smectic	T_C_ (Stability)	Dimension Ǻ		Aspect Ratio (L/D)
						Width (D)	Length (L)	
**2a**	155.3	70.7	54.3	125.0	280.3	7.7	34.6	4.5
**2b**	150.2	78.0	33.2	111.2	261.4	8.34	52.6	6.3
**2c**	149.8	79.3	33.2	112.5	262.3	8.6	57.5	6.7
**2d**	203.9	40.2	57.1	97.3	301.2	8.4	44.0	5.2
**2e**	170.0	66.0	44.0	110.0	280.0	8.3	53.2	6.4
**2f**	167.2	71.8	21.3	93.1	260.3	8.4	66.1	7.9

**Table 6:** FMO Energies e.V. of the prepared compounds **2a** and **2d**.

Compounds	HOMO	LUMO	ΔE
**2a**	−6.52	−3.11	3.41
**2d**	−6.09	−3.07	3.02

## Data Availability

The data presented in this study are available on request from the corresponding author.

## Sample Availability

Samples of the compounds **2(a–f)** are available from the authors.
